# Systemic release of heat-shock protein 27 and 70 following severe trauma

**DOI:** 10.1038/s41598-019-46034-w

**Published:** 2019-07-03

**Authors:** Thomas Haider, Elisabeth Simader, Olaf Glück, Hendrik J. Ankersmit, Thomas Heinz, Stefan Hajdu, Lukas L. Negrin

**Affiliations:** 1 Department of Orthopedics and Trauma Surgery, Medical University of Vienna, Waehringer Guertel 18-20, 1090 Vienna, Austria; 2 Division of Rheumatology, Department of Internal Medicine III, Medical University of Vienna, Waehringer Guertel 18-20, 1090 Vienna, Austria; 3 Department of Thoracic Surgery, Medical University of Vienna, Waehringer Guertel 18-20, 1090 Vienna, Austria; 4 Austrian Research Promotion Agency FFG Projects 852748 and 862068 “APOSEC”, Medical University of Vienna, Waehringer Guertel 18-20, 1090 Vienna, Austria

**Keywords:** Prognostic markers, Prognostic markers

## Abstract

Trauma represents a major cause of morbidity and mortality worldwide. The endogenous inflammatory response to trauma remains not fully elucidated. Pro-inflammation in the early phase is followed by immunosuppression leading to infections, multi-organ failure and mortality. Heat-shock proteins (HSPs) act as intracellular chaperons but exert also extracellular functions. However, their role in acute trauma remains unknown. The aim of this study was to evaluate serum concentrations of HSP 27 and HSP 70 in severely injured patients. We included severely injured patients with an injury severity score of at least 16 and measured serum concentration of both markers at admission and on day two. We found significantly increased serum concentrations of both HSP 27 and HSP 70 in severely injured patients. Concomitant thoracic trauma lead to a further increase of both HSPs. Also, elevated concentrations of HSP 27 and HSP 70 were associated with poor outcome in these patients. Standard laboratory parameters did not correlate with neither HSP 27, nor with HSP 70. Our findings demonstrate involvement of systemic release of HSP 27 and HSP 70 after severe trauma and their potential as biomarker in polytraumatized patients.

## Introduction

Trauma is still a major cause of death accounting for over 5 million deaths globally every year^1^. Severe injuries remain the uncontested number 1 cause of mortality in the population of below 40 years of age^2^. In-hospital mortality remains at a high level of 15%^2^. A common complication in these patients is the development of organ failure. Around 32% develop multiple-organ failure (MOF) during hospitalization. Acute respiratory distress syndrome (ARDS) is one of the most common complications observed in polytraumatized patients with reported rates of up to 50%^2,3^. The endogenous reaction to severe injuries comprises two distinct immunological responses occurring simultaneously. These responses are referred to as systemic inflammation (systemic inflammation response syndrome, SIRS) as well as immunosuppression, commonly referred to as compensatory anti-inflammatory response syndrome (CARS)^4–6^. The lung has been recognized as central actor in orchestrating the endogenous inflammatory response following severe injuries. However, underlying molecular and cellular mediators remain only partially understood^7,8^.

Heat-shock proteins (HSPs) act as intracellular chaperons but are also active extracellularly. These proteins are involved in inhibition of apoptosis, cytoprotection and immunomodulation^9–11^. Serum concentrations of HSPs were previously recognized as potential biomarkers in various diseases including chronic obstructive pulmonary disease (COPD), non-small cell lung cancer, chronic kidney disease, thymus tumours, and stroke among others^9,11–15^. However, their role in the setting of acute trauma remains to be elucidated.

Therefore, in the present study we sought to investigate associations of HSP 27 and HSP 70 serum concentrations following severe injury with concomitant blunt chest trauma, ARDS and mortality.

## Methods

The present study was approved by the local ethics committee of the Medical University of Vienna (no. 368/2011) and complied ethical principals in medical research according to the Declaration of Helsinki.

### Patients

Over a period of 4 years, we prospectively enrolled 120 patients meeting the following inclusion criteria: admission at our urban level I trauma center with severe injuries (injury severity score, ISS above 15) within 1 hour following trauma, primary treatment at the intensive care unit (ICU), and survival of at least 24 hours. We excluded patients with known malignancies and chronic inflammatory lung diseases. Treatment according to standard protocol was not affected by this study. Clinical data, in-hospital mortality and respirator measures were recorded. We used the Berlin classification to determine presence and severity of acute respiratory distress syndrome (ARDS). This classification defines severity in dependence of the Horowitz index (arterial oxygen partial pressure/oxygen fraction in ventilated air) in mild (Horowitz index: 200–299), moderate (Horowitz index: 100–199), and severe (Horowitz index: <100) ARDS^16^. As control group, we recruited 8 healthy volunteers and 5 patients with isolated blunt chest trauma with a median abbreviated injury scale (AIS) of 3 (see Table 1).

**Table 1:** Demographic Characteristics. (ISS = Injury Severity Score, ARDS = Acute respiratory distress syndrome, bold…p-value < 0.05).

Characteristics	Healthy probands n = 8	Isolated thoracic injuries n = 5	Polytraumatized patients n = 120	Polytraumatized patients w/o chest trauma n = 12	Polytraumatized patients with chest trauma n = 108	p-value
Median age (IQR)	36 (20–63)	46 (25–76)	39 (18–85)	35 (18–77)	39 (18–85)	0.484
Male (%)	4 (50)	3 (67)	85 (71)	10 (83)	75 (69)	0.505
Median ISS (range)	—	—	29 (16–59)	28 (16–36)	29 (17–59)	0.194
Mortality (%)	—	0 (0)	5 (4)	0 (0)	5 (5)	0.585
Median AIS (range)	—	3 (3–3)	3 (0–5)	0 (0)	4 (1–5)	**0**.**001**
Severe Thoracic Trauma (%)	—	5 (100)	91 (76)	0 (0)	91 (84)	**0**.**001**
ARDS (%)	—	0 (0)	36 (30)	3 (25)	33 (31)	0.488
Pneumonia (%)	—	0 (0)	35 (29)	6 (50)	29 (27)	0.094
Ventilator days (range)	—	—	3 (0–76)	9 (0–31)	3 (0–76)	0.470

We acquired data presented in this study in a post-hoc analysis of remaining serum samples. Results of data obtained from the same patient collective were previously published^17,18^. In accordance with the local ethics committee of the Medical University of Vienna written informed consent was not obtained since this study involved post-hoc analysis of available serum samples.

### Serum samples and quantification of HSPs

We obtained study-specific serum samples within the first two hours after admission (“day 1”, initial assessment) and between 24 and 48 hours (“day 2”, follow-up assessment) upon admission. Following blood withdrawal and an interval of 15 to 30 minutes to allow thorough coagulation, centrifugation at 3000 g for 15 minutes at room temperature was applied. Serum samples were then aliquoted and stored at −80 °C until measurements.

For quantification of HSP 27 and HSP 70 we used commercially available enzyme linked immunosorbent assays (ELISA) provided by R&D Systems (Minnesota, USA, DuoSet IC). Analyses were performed according to the manufacturer’s brochure. We performed all measurements in technical duplicates.

### Statistics

The software basis of data analyses and visualization were IBM SPSS Statistics 24.0 (IBM, Armonk, NY, USA) and GraphPad Prism 5 (GraphPad Software, La Jolla, CA, USA). For comparison of metrical and ordinal values between 2 independent groups the non-parametrical Mann-Whitney U-test was utilized. We used the Wilcoxon rank-sum test for comparison of metrical and ordinal values between connected samples and the Kruksal-Wallis test to compare metric and ordinal variables between 3 independent groups. The chi-square test was used for comparison of nominal variables. For correlation of non-parametric metric variables Spearman’s rank correlation coefficient test was utilized. Data are given and plotted as mean ± standard error of the mean (SEM) if not stated otherwise. Following median values interquartile ranges are given in brackets. A p-value < 0.05 was considered statistically significant.

## Results

### Demographics

The patient collective consisted of 120 severely injured patients. The majority of patients were male (n = 85, 70.8%). The median age was 39 (27–55) years. The patients presented with a median ISS of 29 (22–38) at our trauma unit. Most patients (n = 108, 90.0%) suffered from concomitant blunt chest trauma with median AIS of 3 (3–4). Severe chest trauma (AIS ≥ 3) was observed in 91 patients (75.8%). A total of 50 patients (41.7%) developed pulmonary complications, including 36 patients (30%) sustaining ARDS and 35 patients (29.2%) suffering from pneumonia, of which 21 patients (17.5%) developed both ARDS and pneumonia. The in-hospital mortality rate was 4.2% (n = 5). The median hospitalization time was 26 days (17–55) of which a median of 10 days (3–18) were spent at the ICU. The median ventilator days were 3 (1–10). The median ISS was comparable among patients with and patients without chest trauma (29 vs. 28, p = 0.19). In both deceased patients and patients developing pulmonary complications the median ISS was higher compared to surviving patients and patients with no pulmonary complications, respectively (57 vs. 29, p < 0.01; 34 vs. 27, p < 0.01).

### Polytrauma leads to increased HSP 27 and HSP 70 serum concentrations

**Figure 1:**
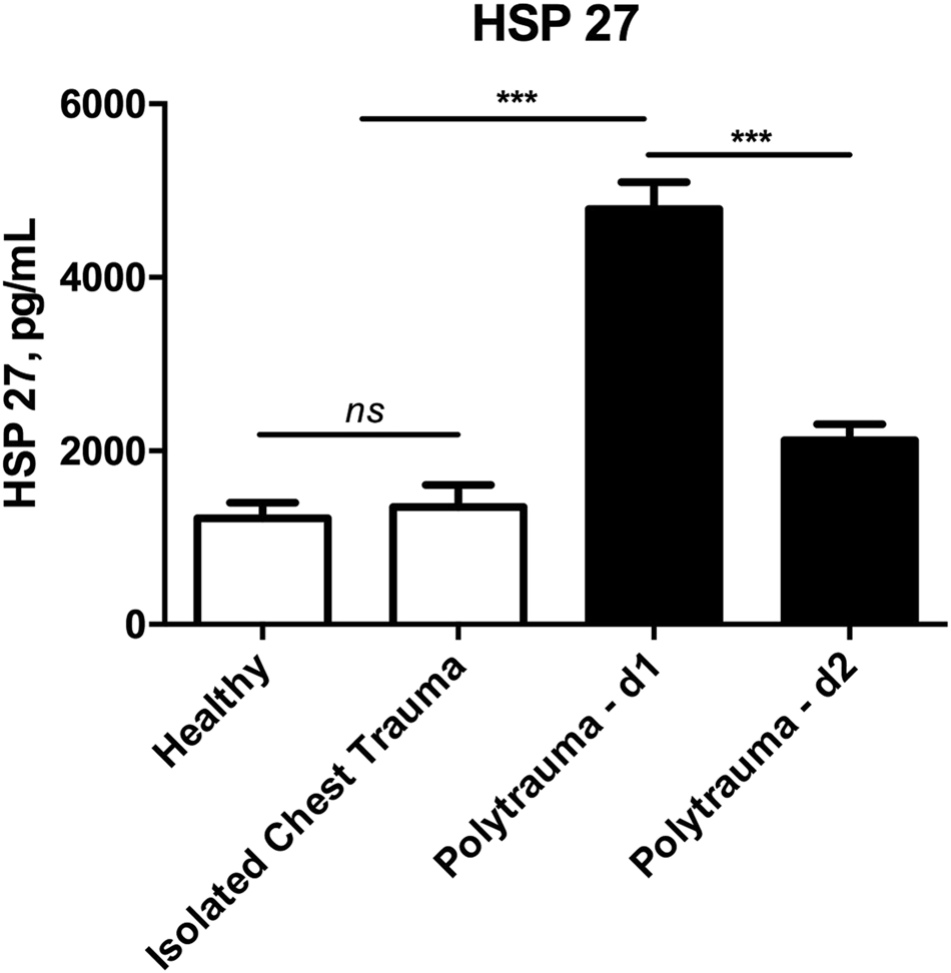
Comparison of serum concentrations of Heat shock protein 27 (HSP 27) between healthy controls, patients with isolated injuries and polytraumatized patients. (ns…not significant, ***p < 0.001).

**Figure 2:**
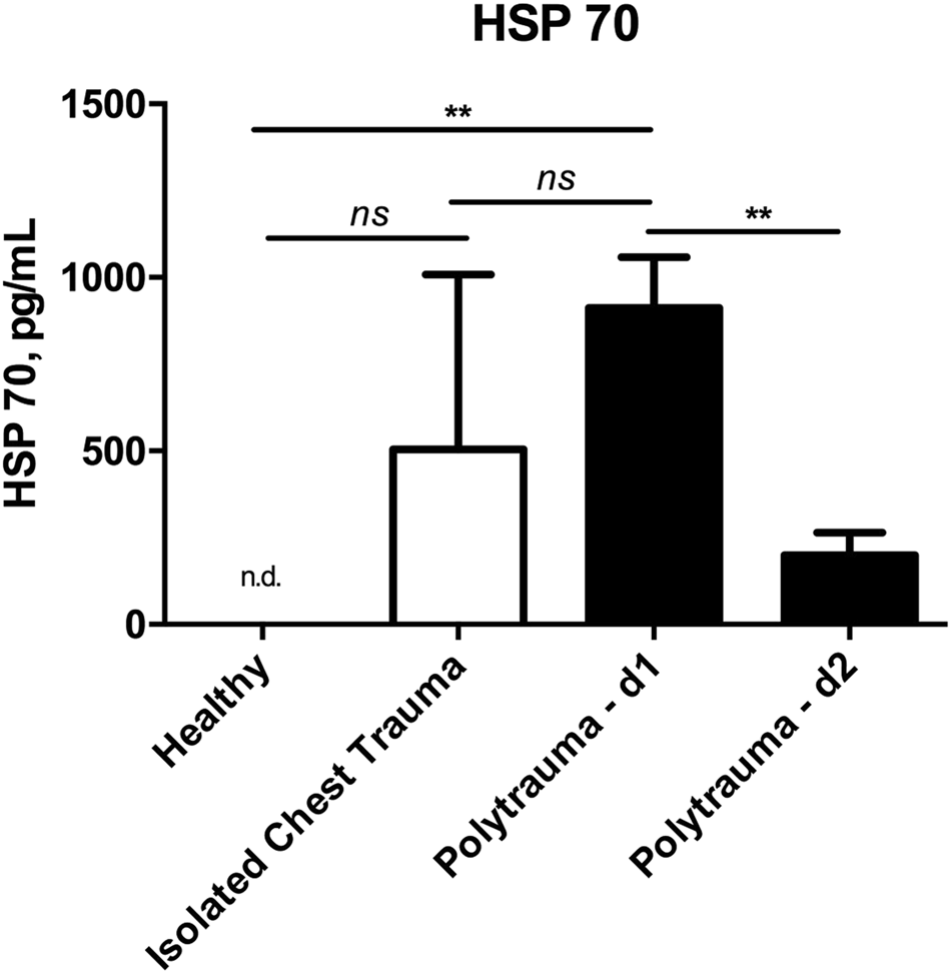
Comparison of serum concentrations of Heat shock protein 70 (HSP 70) between healthy controls, patients with isolated injuries and polytraumatized patients. (n.d….not detectable, ns…not significant, **p < 0.01, ***p < 0.001).

Compared to healthy controls, severe injuries lead to significantly increased serum levels of HSP 27 and HSP 70 at day 1. Compared to isolated blunt chest trauma, serum of concentrations of HSP 27 but not of HSP 70 were significantly elevated at day 1. (Figs 1 and 2) Both cytokines significantly decreased in the group of polytraumatized patients from day 1 to day 2 (HSP 27 - day 1 vs. day 2: 4454 ± 296 vs. 2034 ± 168 pg/mL, p < 0.001; HSP 70 - day 1 vs. day 2: 835 ± 134 vs. 197 ± 61 pg/mL, p < 0.001) (Figs 1 and 2).

We observed weak correlation between overall injury severity as indicated by the ISS and serum concentrations of HSP 27 and HSP 70 (HSP 27 - d1: r = 0.354, p < 0.001; HSP 27 - d2: r = 0.241, p < 0.01; HSP 70 - d1: r = 0.394, p < 0.001; HSP 70 - d2: r = 0.208, p < 0.05).

### Concomitant thoracic trauma further increases serum concentrations of HSP 27 and 70 in polytraumatized patients

**Figure 3:**
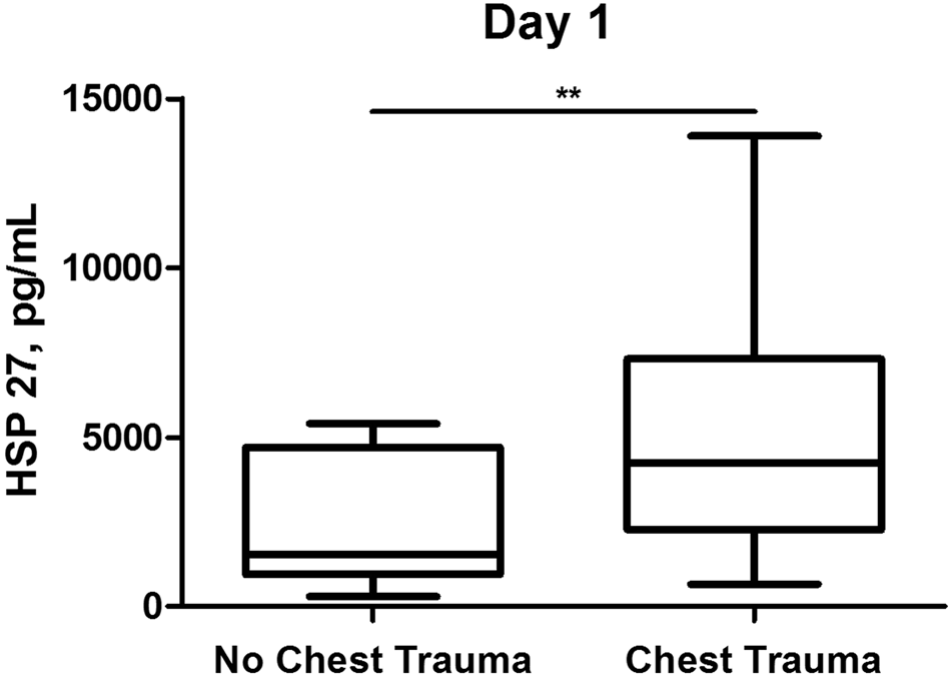
Comparison of serum concentrations of Heat shock protein 27 (HSP 27) between polytraumatized patients with and polytraumatized patients without concomitant blunt chest trauma at day 1. (**p < 0.01).

**Figure 4:**
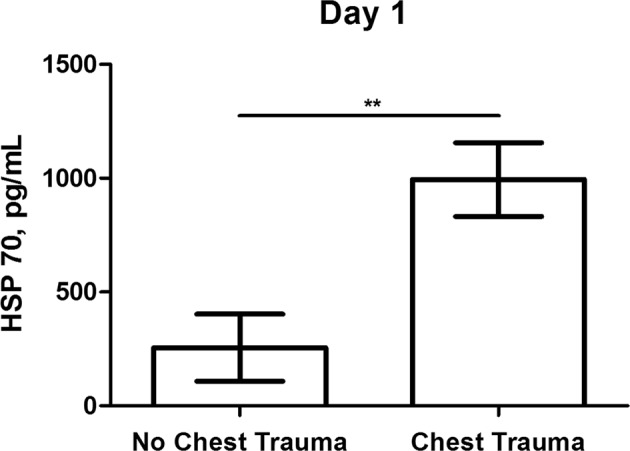
Comparison of serum concentrations of Heat shock protein 70 (HSP 70) between polytraumatized patients with and polytraumatized patients without concomitant blunt chest trauma at day 1. (**p < 0.01).

At initial assessment both HSP 27 and HSP 70 serum concentrations were significantly higher in polytraumatized patients sustaining concomitant blunt chest trauma (HSP 27: 5050 ± 331 vs. 2581 ± 567 pg/mL, p < 0.01; HSP 70: 994 ± 162 vs. 255 ± 148 pg/mL, p < 0.01). (Figs 3 and 4) At follow-up measurement both HSP 27 and HSP 70 serum concentrations in patients with and patients without chest trauma approximated (HSP 27: 2142 ± 178 vs. 1872 ± 1164 pg/mL, p < 0.05; HSP 70: 191 ± 67 vs. 254 ± 254 pg/mL, p = 0.566).

ROC-analysis revealed prognostic values for HSP 27 and HSP 70 serum concentrations for the detection of concomitant chest injury in the study population of polytraumatized patients (HSP 27 - d1: AUC: 0.744, 95% CI: 0.546–0.941, p < 0.05; HSP 27 - d2: AUC: 0.793, 95% CI: 00.580–1.000, p < 0.01; HSP 70 - d1: AUC: 0.778, 95% CI: 0.618–0.938, p < 0.05). For day 1 HSP 27 serum concentrations a cut-off value of 1165 pg/mL reached 92.3% sensitivity and 62.5% specificity, while for day 1 HSP 70 serum concentrations, a cut-off value of 50 pg/mL reached 69.2% sensitivity and 87.5% specificity for detection of chest trauma in polytraumatized patients.

Only weak correlations between HSP 27 and HSP 70 serum concentrations and chest injury severity as indicated by the AIS was observed (HSP 27 - d1: r = 0.278, p < 0.01; HSP 27 - d2: r = 0.215, p < 0.05; HSP 70 - d1: r = 0.271, p < 0.01; HSP 70 - d2: r = −0.024, p = 0.406).

### Increased serum concentrations of HSP 27 and 70 are associated with worse outcome

**Figure 5:**
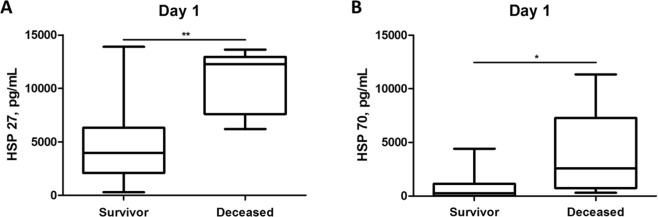
Comparison of day 1serum concentrations of (**A**) Heat shock protein 27 (HSP 27) and (**B**) Heat shock protein 70 (HSP 78) between polytraumatized deceased patients and polytraumatized surviving patients. (*p < 0.05, **p < 0.01).

We found significantly increased HSP 27 and HSP 70 serum concentrations in deceased patients compared to surviving patients (HSP 27 - day 1: 10670 ± 1356 vs. 4520 ± 294 pg/mL, p < 0.01; HSP 27 - day 2: 9261 ± 2817 vs. 1982 ± 155 pg/mL, p < 0.01; HSP 70 - day 1: 3717 ± 1971 vs. 776 ± 110 pg/mL, p < 0.05; HSP 70 - day 2: 2788 vs. 170 ± 59 pg/mL, p < 0.05). (Fig. 5) Serum concentrations of HSP 27 and HSP 70 at initial assessment were of prognostic value predicting mortality in our patient collective (HSP 27 - d0: AUC: 0.911, 95% CI: 0.818–1.000, p < 0.01; HSP 70 - d1: AUC: 0.822, 95% CI: 0.662–0.982, p < 0.05). For HSP 27 concentrations at day 1, a cut-off value of 6000 pg/ml showed 100% sensitivity and 73.3% specificity for mortality prediction. We found at a cut-off value of 1000 pg/ml of HSP 70 serum concentration at day 1 a sensitivity of 80% and a specificity of 73.3% for prediction of mortality in these patients.

Also, patients developing ARDS showed higher serum concentrations of HSP 27 on day 2 and of HSP 70 on day 1 compared to patients without ARDS development during hospitalization (HSP 27 - day 2: 2685 ± 382 pg/mL vs. 1896 ± 204 pg/mL, p < 0.05; HSP 70 - day 1: 1442 ± 367 pg/mL vs. 658 ± 117 pg/mL, p < 0.05).

No difference was found in HSP 27 and HSP 70 serum concentrations in patients developing pneumonia compared to patients without pneumonia (data not shown).

### HSP 27 and 70 serum concentrations do not correlate with standard laboratory parameters

Overall, we found no relevant correlation with standard laboratory parameters including hemoglobin concentration, leukocyte count, and CRP-concentration. The respective correlations are given in detail in Supplementary Table 1.

## Discussion

In the present study we demonstrate that severe injuries are associated with increased HSP 27 and HSP 70 serum concentrations. Our data suggests that concomitant thoracic trauma induces further increment of these proteins in the serum of polytraumatized patients. Higher serum concentrations of both HSP 27 and HSP 70 were associated with the development of ARDS and mortality.

Severe injuries induce a strong endogenous inflammatory response in the acute phase. In previous studies, multiple systemic cytokines were shown to be involved in this pro-inflammatory state including Interleukin (IL-)-1, IL-6, IL-8, and TNF-α (tumor necrosis factor-α) among others. In case of overwhelming inflammatory signals SIRS develops with high rates of morbidity and mortality^19,20^. Current concepts of SIRS and CARS suggest co-existence of both immunological phenomena immediately after trauma. Immunosuppression and lymphocyte dysfunction leads to increased susceptibility to infections and sepsis^5,6^. Recent studies show that the presence of extracellular HSP 27 induces a shift towards anti-inflammatory pathways in macrophages via NF-κB (nuclear factor-κB)^10,21^. Interestingly, Li *et al*. showed in 2011 that NF-κB activity is up-regulated in monocytes of polytraumatized patients 24 hours after injury^22^. We found higher serum concentrations of HSP 27 also within the first 24 hours after injury compared to day 2. Increased serum concentrations of HSP might be involved in high NF-κB activity after severe trauma as reported by Li *et al*.^22^.

At cellular level, neutrophil granulocytes represent the first-line of response to trauma^20^. They produce reactive oxygen species (ROS) as defense mechanism against invading microorganisms. Also, hypoxia and reperfusion lead to production of ROS. This ROS overload causes secondary tissue and organ damage potentially resulting in multiple organ failure (MOF), the most common cause of death in the sub-acute and late phase after polytrauma^19,20^. HSP 27 was shown to be capable of regulating redox hemostasis^9,23^. The early systemic release in serum HSP 27 following severe trauma could represent an endogenous counteract to circumvent damage caused by ROS in the acute phase after trauma.

Intracellular HSPs act as chaperons preventing protein misfolding in presence of stressors. Aside from this well-known property, many other functions such as inhibition of apoptosis, cytoskeleton stabilization, support of hormone regulation, among others have been proposed^10,24^. Cell death leads to passive release of cytosolic HSPs. But also, active secretion of HSPs has been described^24–26^. The mode of release, either by cell death or active secretion, seems to be defining the effects of HSPs on the immune system. Passive release following necrotic death leads to pro-inflammatory characteristics of extracellular HSPs, while actively secreted HSPs cause immunosuppression^10,24,27^. This diverse role of HSPs seems to be a result of concomitant released factors influencing the cellular environment and their response to extracellular HSPs. One way of active secretion of HSPs is via exosome release. It was reported that composition of exosomes determines the response to HSPs^24^. This Janus-head characteristic is interesting in the trauma setting.

The lung is recognized as central organ in immunity in health and disease^28^. Direct chest trauma leads to marked alterations of expression of inflammatory proteins and was shown to be associated with early development of ARDS^3,8^. We show that concomitant thoracic trauma in polytraumatized patients is associated with further increase of HSP serum concentrations. Also, we found increased concentrations in patients who developed ARDS. Our data indicate that extracellular HSPs might be involved in the connection of chest trauma and development of ARDS. Previous work shows that NF-κB is elevated in the lung following thoracic injury^8^. As mentioned above, previous data published by Salari *et al*. show a direct connection between extracellular HSPs and elevated NF-κB expression^10^. Also, transient hypoxia in healthy individuals was shown to be capable of inducing increased HSP serum concentrations^29^. Taken together, these previous findings corroborate our results that lung trauma causes increment of serum HSP 27 and HSP 70.

Even though the number of deceased patients in our study population was low, we think that our findings further make a case for HSPs as biomarkers for predicting mortality in polytraumatized patients. In our opinion, extracellular HSPs might be involved in development of early and late MOF caused by either SIRS or CARS. Early death is mainly caused by exsanguation or traumatic brain injury. We excluded patients with mortality within the first 24 hours to avoid these causes as potential bias.

In 2015, a small pilot study with 18 patients showed an increase of HSP 70 serum concentrations following severe injuries with peak levels immediately after trauma and gradual reduction^30^. These findings are in line with our observations. Hashiguchi *et al*. showed in 2001 that trauma elevates expression of HSPs in leukocytes^31^. Based on this report, leukocytes might be one of the major cellular origins of elevated HSPs serum concentrations following severe trauma. Flohé *et al*. demonstrated that soft-tissue trauma during orthopedic surgery is capable to induce local increment of HSPs concentrations further supporting our data^32^. Further corroborating our findings, Pittet *et al*. showed that HSP 72, a synonym for HSP70, is systemically elevated following severe trauma^33^. On the other hand, they report decreased levels in non-surviving patients^33^. Their sole measurement occurred at 12 to 48 hours after admission. We suggest that this broad time frame might have caused this difference in observed results since HSP concentrations were reported to markedly decrease within the first days as this was also shown in the present study. Also, other studies have demonstrated worse outcomes with increased serum concentrations of HSP70 in other clinical entities including sepsis^30,34,35^.

Serum concentrations of HSPs are under investigation as potential biomarkers in several fields^11–15,36,37^. Based on previous and presented results, recent trauma in patients´ medical history needs to be carefully considered as confounder of HSP serum concentrations.

Our study has some limitations. We did not account for preexisting conditions in our patient cohort. Especially inflammatory lung diseases among others could have interfered with our results. Furthermore, our patient collective of deceased patients is comparatively small which might has confounded our results regarding correlation with mortality.

In conclusion, our study provides further insight in heat-shock response following severe trauma. Compared to healthy individuals and patients with isolated injuries, increased serum concentrations of HSP 27 and HSP 70 were demonstrated. We showed that concomitant blunt chest trauma is able to further induce an increment of these proteins in the circulation. Higher concentrations were associated with mortality and the development of ARDS.

## Supplementary information

Supplementary Dataset 1
