# Supplementary material for: Systemic release of heat-shock protein 27 and 70 following severe trauma
Source: Sci Rep. 2019 Jul 3;9:9595. doi: 10.1038/s41598-019-46034-w (PMC6610099; doi:10.1038/s41598-019-46034-w)
Supplement: Supplementary file 1 — Supplementary Dataset 1 [file 41598_2019_46034_MOESM1_ESM.pdf]

Systemic release of heat-shock protein 27 and 70 following severe trauma.

Thomas Haider[1*], Elisabeth Simader[2], Olaf Glück[2], Hendrik J. Ankersmit[2], Thomas Heinz[1], Stefan Hajdu[1], Lukas L. Negrin[1]

1 Department of Orthopedics and Trauma Surgery, Medical University of Vienna, Waehringer Guertel 18-20, 1090 Vienna, Austria

2 Department of Thoracic Surgery, Medical University of Vienna, Waehringer Guertel 18-20, 1090 Vienna, Austria

* Corresponding Author: Thomas Haider, M.D., Ph.D.

Department of Orthopedics and Trauma Surgery

Medical University of Vienna

Waehringer Guertel 18-20

1090 Vienna, Austria

email: thomas.a.haider@meduniwien.ac.at

			HSP27_d1	HSP27_d2	HSP70_d1	HSP70_d2	CRP_d1	CRP_d2	Leukos_d1	Leukos_d2	HB_d1	HB_d2
Spearman-Rho	HSP27_d1	Correlation coefficient	1,000	,350**	,769**	,059	-,119	,195*	,076	-,004	-,296**	-,118
		Sig. (2-seitig)	.	,000	,000	,579	,209	,045	,421	,964	,001	,227
		N	114	100	106	92	113	107	114	107	114	107
	HSP27_d2	Correlation coefficient	,350**	1,000	,324**	,243*	-,037	,159	-,079	-,049	-,436**	-,193
		Sig. (2-seitig)	,000	.	,001	,019	,706	,115	,426	,628	,000	,054
		N	100	105	95	93	104	99	105	99	105	100
	HSP70_d1	Correlation coefficient	,769**	,324**	1,000	,193	-,170	,136	,131	,035	-,185	-,014
		Sig. (2-seitig)	,000	,001	.	,067	,079	,175	,176	,729	,056	,888
		N	106	95	108	91	107	101	108	101	108	102
	HSP70_d2	Correlation coefficient	,059	,243*	,193	1,000	,095	-,159	,230*	,043	-,205*	-,029
		Sig. (2-seitig)	,579	,019	,067	.	,359	,131	,024	,687	,043	,781
		N	92	93	91	97	96	92	97	92	97	93
	CRP_d1	Correlation coefficient	-,119	-,037	-,170	,095	1,000	,374**	,052	,058	,087	,078
		Sig. (2-seitig)	,209	,706	,079	,359	.	,000	,573	,546	,349	,414
		N	113	104	107	96	119	111	119	111	119	112
	CRP_d2	Correlation coefficient	,195*	,159	,136	-,159	,374**	1,000	,118	,060	-,054	-,152
		Sig. (2-seitig)	,045	,115	,175	,131	,000	.	,216	,526	,570	,110
		N	107	99	101	92	111	112	112	112	112	112
	Leukos_d1	Correlation coefficient	,076	-,079	,131	,230*	,052	,118	1,000	,228*	,243**	,046
		Sig. (2-seitig)	,421	,426	,176	,024	,573	,216	.	,016	,008	,631
		N	114	105	108	97	119	112	120	112	120	113
	Leukos_d2	Correlation coefficient	-,004	-,049	,035	,043	,058	,060	,228*	1,000	,154	,354**
		Sig. (2-seitig)	,964	,628	,729	,687	,546	,526	,016	.	,104	,000
		N	107	99	101	92	111	112	112	112	112	112
	HB_d1	Correlation coefficient	-,296**	-,436**	-,185	-,205*	,087	-,054	,243**	,154	1,000	,461**
		Sig. (2-seitig)	,001	,000	,056	,043	,349	,570	,008	,104	.	,000
		N	114	105	108	97	119	112	120	112	120	113
	HB_d2	Correlation coefficient	-,118	-,193	-,014	-,029	,078	-,152	,046	,354**	,461**	1,000
		Sig. (2-seitig)	,227	,054	,888	,781	,414	,110	,631	,000	,000	.
		N	107	100	102	93	112	112	113	112	113	113
